# Decision-Making in Unilateral Progressive Condylar Growth of the Mandible: Biological Insights and the Role of Proportional Condylectomy

**DOI:** 10.3390/jcm15124654

**Published:** 2026-06-16

**Authors:** Sergio Olate, Victor Ravelo, Marcelo Parra, Majeed Rana

**Affiliations:** 1 Department of Oral Diagnosis, Division of Oral and Maxillofacial Surgery, State University of Campinas, Piracicaba 13414-903, SP, Brazil; 2 PhD Program & Center for Research in Morphology and Surgery, Faculty of Medicine, Universidad de La Frontera, Temuco 4811230, Chile; victor.ravelo.s@gmail.com (V.R.);; 3 GIPO, Facultad de Ciencias de la Salud, Universidad Autónoma de Chile, Temuco 4780000, Chile; 4 Department of Adult Dentistry, Faculty of Dentistry, Universidad de La Frontera, Temuco 4811230, Chile; 5 Department of Oral and Maxillofacial Surgery, Faculty of Dentistry, National University of Singapore, National University Centre for Oral Health, Singapore 119085, Singapore; 6 Department of Oral and Plastic Maxillofacial Surgery, Albert Einstein University Hospital Ulm, Albert-Einstein-Allee 11, 89081 Ulm, Germany

**Keywords:** unilateral condylar hyperplasia, condylar osteochondroma, condylectomy, facial asymmetry

## Abstract

**Background:** Unilateral progressive condylar growth (UPCG) represents a complex clinical condition characterized by abnormal enlargement of the mandibular condyle, leading to progressive facial asymmetry and functional impairment. **Objectives:** The aim of this review is to analyze the biological, clinical, and therapeutic factors guiding condylectomy, assess the current role and scope of proportional condylectomy, and propose an algorithm to guide its indication in patients with UPCG. **Methods:** A narrative review was conducted to analyze the biological, clinical, and therapeutic factors involved in the indication for condylectomy in patients with progressive unilateral condylar growth. Studies including patients diagnosed with unilateral condylar hyperplasia or condylar osteochondroma who underwent surgical treatment were considered to evaluate clinical indications, timing of intervention, and outcomes. Special attention was given to the concept of proportional condylectomy. **Results:** Current evidence indicates that early intervention may control disease progression, reduce the severity of residual deformity, and minimize the need for secondary orthognathic surgery. The integration of clinical findings, three-dimensional imaging, and patient-specific factors is essential for appropriate treatment planning. **Conclusions:** Based on these considerations, a structured clinical algorithm is proposed to guide decision-making in patients with unilateral progressive condylar growth. This approach supports individualized treatment strategies aimed at optimizing functional and esthetic outcomes.

## 1. Introduction

Condylectomy of the mandibular condyle has historically been described as a technique for controlling pathological condylar growth, with several variations [1,2,3,4]. Unilateral condylar hyperplasia (UCH) and temporomandibular joint (TMJ) osteochondroma (OC) are two entities that share a progressive and uncontrolled growth of the mandibular condylar head, resulting in asymmetric volumetric enlargement compared to the contralateral condyle and a progressive facial deformity with asymmetry, frequently self-limited at an undetermined age and stage. Their clinical presentation is variable and depends on multiple individual factors, making their management a continuous challenge for the maxillofacial surgeon [5,6,7].

From an epidemiological perspective, UCH predominantly occurs in females, with higher frequency during adolescence, whereas OC is more commonly observed in adults [5,8]. Both UCH and OC represent clear models of progressive non-neoplastic growth that may become potentially devastating, with significant impact not only from a functional and esthetic standpoint but also in psychological and social domains [9].

The diagnosis of active disease has been widely debated. To date, no specific systemic or hereditary conditions have been associated with the origin of the disease, contributing to the difficulty of early diagnosis [7]. Various diagnostic tools have been proposed, including serial two-dimensional imaging follow-up, three-dimensional tomographic studies, bone scintigraphy using SPECT, and other functional techniques, aiming to identify the progression of condylar growth and guide therapeutic decision [10]. SPECT is considered a useful tool; however, some studies report low specificity and sensitivity as a diagnostic method [11], suggesting that the integration of multiple methods and tools may represent the best approach.

Determining condylar activity and growth rate is one of the key elements in therapeutic planning, although longitudinal behavior shows that osteoblastic activity may remain increased or stabilize over time [12]. This variability reinforces the need for clinical correlation and tomographic imaging in any therapeutic decision.

In this context of clinical, diagnostic, and therapeutic variability, condylectomy represents a real and reproducible alternative for the management of patients with active unilateral progressive condylar growth (UPCG) and progressive facial asymmetry, demonstrating favorable clinical outcomes from the early stages in halting the process [13,14]. However, the correct indication of the most appropriate type of condylectomy for each patient remains a matter of debate.

The aim of this review is to analyze the biological, clinical, and therapeutic factors involved in the indication of condylectomy, to evaluate the current status of proportional condylectomy by identifying its clinical and conceptual scope, and, finally, to propose an algorithm for guiding the indication of condylectomy as a therapeutic strategy in patients with UPCG.

## 2. Materials and Methods

This narrative review was conducted to analyze the biological, diagnostic, clinical, and therapeutic factors involved in the management of unilateral progressive condylar growth (UPCG), with special emphasis on the current role and clinical application of proportional condylectomy.

A literature search was performed using PubMed/MEDLINE, Scopus, and Google Scholar databases to identify relevant publications related to unilateral condylar hyperplasia, condylar osteochondroma, condylectomy, proportional condylectomy, mandibular asymmetry, temporomandibular joint surgery, and condylar growth. The search included articles published in English up to January 2026. Additional references were manually identified from the reference lists of selected articles.

The review was focused on studies addressing biological mechanisms of condylar growth, diagnostic criteria, imaging evaluation, surgical management, proportional condylectomy techniques, postoperative orthodontic management, and treatment outcomes in patients with UPCG. Original articles, retrospective and prospective clinical studies, systematic reviews, and relevant historical publications were considered according to their conceptual and clinical contribution to the topic.

Because this study was designed as a narrative review, a formal systematic review protocol, PRISMA strategy, or quantitative meta-analysis was not performed. The selection of the literature was based on the relevance, scientific impact, methodological consistency, and historical significance of the publications in relation to the objectives of the review.

Case reports with isolated observations, animal studies, duplicated publications, and articles without direct relevance to clinical decision-making or proportional condylectomy were excluded from the qualitative synthesis.

The final analysis was structured to integrate biological concepts, clinical presentation, imaging findings, surgical evolution, and therapeutic algorithms to support a contemporary decision-making model for UPCG management.

Conceptual schematic illustrations included in the manuscript were created using design software (Canva, online version) and subsequently refined with AI-assisted visual support tools (ChatGPT, GPT 5.5, OpenAI, San Francisco, CA, USA) exclusively for graphical organization and visual enhancement. The authors entirely developed all scientific concepts and anatomical relationships and performed final figure validation.

## 3. Results

### 3.1. Condylar Development and Growth in Children and Adolescents

The mandibular condyle represents an essential osteochondral unit in the growth and development of the facial skeleton. Its contribution to mandibular growth largely determines the vertical and sagittal configuration of the maxillomandibular complex. From a structural perspective, condylar size and morphology are influenced by genetic and epigenetic variables, including sex, skeletal maturation, and systemic factors. The microstructural quality of the condyle has been associated with polymorphisms in genes related to calcium and vitamin D metabolism, particularly VDBP and PTH [15], indicating that subchondral architecture depends on local stimuli, endocrine regulation, and integrated genetic control.

It is not possible to define a universal size or volume for the mandibular condyle. Its position and dimensions are significantly associated with facial skeletal morphology and maxillomandibular relationships [16]. Three-dimensional studies have confirmed that condylar height and ramus dimensions vary systematically according to vertical growth patterns [17,18], reinforcing the relationship between condylar activity and facial morphology.

Unlike most of the facial skeleton, which grows predominantly through intramembranous ossification, the mandibular condyle develops via endochondral ossification. This biological characteristic results in a stratified cellular organization, including proliferative zones, hypertrophic chondrocytes, and regions of active ossification. Molecular regulation of this process involves the Wnt/β-catenin pathway and the activity of Gli1+ progenitor cells in subchondral bone [19], mechanisms that control the balance between chondrogenic proliferation and osteogenic differentiation.

Condylar cartilage is a biologically active tissue. The presence of fibrocartilaginous stem cells with self-renewal capacity and the ability to differentiate into chondrogenic and osteogenic lineages [20] demonstrates the biological plasticity during childhood and adolescence. This cellular component explains the adaptive capacity of the condyle in response to functional stimuli.

The macroscopic changes correlate with microstructural modifications. Trabecular reorganization and increased bone density described in histomorphometric studies [21] indicate that condylar growth involves active subchondral remodeling.

In this context, the biomechanical environment plays a critical regulatory role. Experimental models have demonstrated that functional overload modifies the thickness of the hypertrophic chondrocyte layer and alters endochondral ossification [22], also suggesting that mechanical stimuli may modify growth trajectories. Additional studies have confirmed that variations in loading or overloading affect bone volume and trabecular thickness of the condyle [22], supporting the application of mechanistic theory in mandibular growth.

Tissue response, therefore, depends on the magnitude of the stimulus. Activation of the FGF9/FGFR3 pathway is associated with adaptive compensatory remodeling, whereas activation of Rac2–p38 MAPK is linked to degenerative changes [23]. This balance between anabolic and catabolic pathways defines the adaptive capacity of the growing condyle.

Mechanical regulation also interacts with the hormonal environment. Estrogen modulates the expression of type I and III collagen in cells derived from condylar cartilage under mechanical loading [24], suggesting a mechano-hormonal integration in extracellular matrix maturation. However, short-term occlusal modifications do not produce significant changes in the growing condyle [25], indicating that structural remodeling requires sustained stimuli.

From a morphological perspective, three-dimensional imaging studies have demonstrated variations in mandibular architecture according to skeletal pattern [18], while condylar axis angulation has been associated with functional adaptation and mandibular morphology [26]. Other studies have also identified clear differences in linear measurements at the condylar level according to specific mandibular morphological conditions [18].

Longitudinal growth of the mandibular ramus has been quantified using three-dimensional imaging, demonstrating progressive increases in condylar height and ramus dimensions until skeletal maturity [27]. Between the ages of 10 and 15 years, significant vertical growth is observed (ranging from 10 to 15 mm in height), with documented sex differences and association with mandibular rotation [28]. Concurrently, mandibular corticalization progresses rapidly during adolescence, increasing from approximately 20% at age 11 to over 80% structural consolidation by ages 15–17 [29].

During adolescence, inflammatory processes may disrupt condylar homeostasis. For example, in patients with osteoarthritis associated with disk displacement, both quantitative and qualitative changes in condylar morphology have been documented following conservative treatment [30]. The involvement of inflammatory mediators and bone remodeling mechanisms in the temporomandibular joint [31] further supports the integration between tissue biology and functional environment, suggesting that early orthopedic intervention may influence condylar asymmetry during mixed dentition [32].

**Figure 1:**
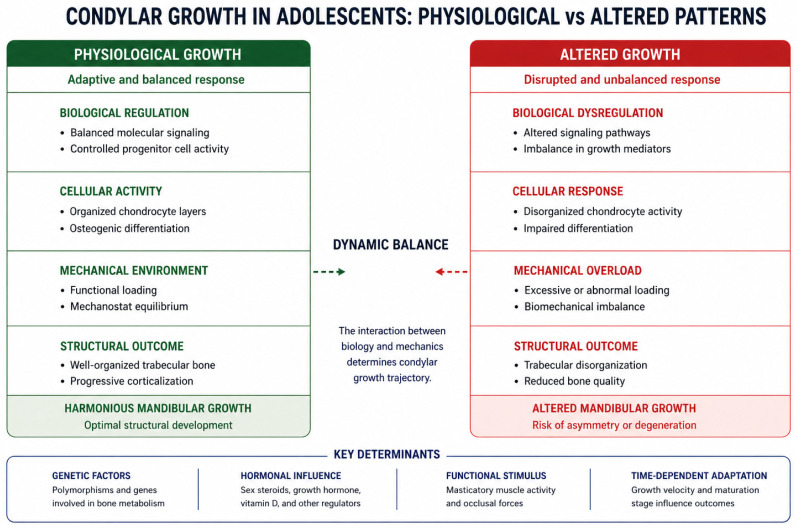
Highlighting the transition from adaptive physiological remodeling to progressive pathological asymmetry. This schematic illustration was created using design software (Canva, online version) and subsequently processed and enhanced in ChatGPT (OpenAI), and was then reviewed and validated by the authors. The arrows show the dynamic balance between processes.

Overall, condylar growth in children and adolescents should be understood as the result of a coordinated interaction between endochondral ossification, genetic regulation, subchondral molecular signaling, chondrogenic cellular activity, load-dependent mechanical stimuli, hormonal modulation, and functional influence (Figure 1). This biological integration determines the structural configuration of the maxillomandibular complex during adolescence and explains the morphological variability observed in craniofacial development.

### 3.2. Unilateral Condylar Hyperplasia and Osteochondroma as Models of Unilateral Progressive Condylar Growth (UPCG)

Unilateral condylar hyperplasia (UCH) is defined as an abnormally increased progressive growth of one mandibular condyle relative to the contralateral side. The increase in condylar size is commonly associated with hemilateral growth of the mandibular structure [33].

UCH primarily occurs during adolescence and is typically characterized by an elongated condyle, contralateral crossbite, transverse chin deviation, and a tendency toward a Class III sagittal relationship [34].

Another similar model of benign condylar growth that may also produce significant asymmetric changes is osteochondroma (OC) [35]. Several studies have analyzed the growth process in both UCH and OC, demonstrating differences in growth rate [36], although without identifying clear histological differences in terms of cellular elements or the expression of specific markers [37].

In the case of OC, clinical presentation is more frequently observed in individuals over 18 years of age (adults), characterized by a condylar morphology with multidirectional growth. It commonly presents as facial asymmetry with unilateral vertical growth, chin deviation, and open bite on the affected side, and may be associated with either Class I or Class III sagittal patterns [38].

Based on these findings, it may be proposed that the microstructural model and potentially the initiating stimulus of benign progressive condylar growth could share similarities in both entities, although their clinical behavior and morphological characteristics appear to differ considerably [39].

Thus, patient age and the degree of mandibular skeletal maturity may play a relevant role in the clinical characteristics of each condition [40,41]. Consequently, skeletal growth status could influence clinical presentation, considering that OC in adults is generally observed after growth has ceased, whereas UCH predominantly occurs in individuals with active growth.

Three-dimensional analysis frequently demonstrates that in cases of UPCG there may be elongation of the mandibular ramus, increased condylar volume, and significant structural asymmetry [42,43]. 3D analysis in patients with UCH has also suggested unstable trajectories and altered lateral pterygoid muscle activity, even when maximum mouth opening is not severely limited [44]. Increased condylar volume may show a tendency toward a Class III pattern [45]. In addition, sustained biomechanical stimuli could potentially influence condylar growth, as the trabecular architecture of the mandibular condyle demonstrates orientation adapted to functional loading, with anisotropic organization and greater bone volume fraction in superior regions, reflecting structural biomechanical adaptation [46].

Under physiological conditions, the condylar head is organized into fibrous, proliferative, pre-hypertrophic, and hypertrophic layers, which progress through endochondral ossification. Mechanical loading appears to modulate this process by increasing cellular proliferation and the expression of chondrogenic and osteogenic markers, together with increased cartilage thickness [47].

UCH is histologically characterized by increased thickness of the proliferative and fibrous layers, variable presence of subchondral cartilaginous islands, and structural heterogeneity, which may complicate uniform characterization [37,48]. From a molecular perspective, increased expression of growth factors such as IGF-1, BMP-2, and TGF-β1 has been observed, particularly in proliferative and hypertrophic layers [38,49], suggesting possible anabolic activation of the endochondral process.

OC shares a histological pattern based on active endochondral ossification, with the presence of a cartilage cap and medullary continuity with the underlying bone [50]. As in UCH, chondrocyte proliferation and progressive bone formation are observed. Histopathological comparison has demonstrated similarities in layered organization and expression of osteogenic markers, although OC tends to exhibit a thicker cartilage cap and a higher proliferative index [36,51].

From a structural perspective, micro-CT analysis in condylar hyperplasia has not demonstrated marked trabecular differences between clinical types [52], while molecular studies have not identified classical tumor-associated somatic mutations in UCH [53]. These findings may suggest that although OC presents a more organized and proliferative pattern, both entities could share biological mechanisms associated with cartilaginous expansion and active endochondral ossification.

**Figure 2:**
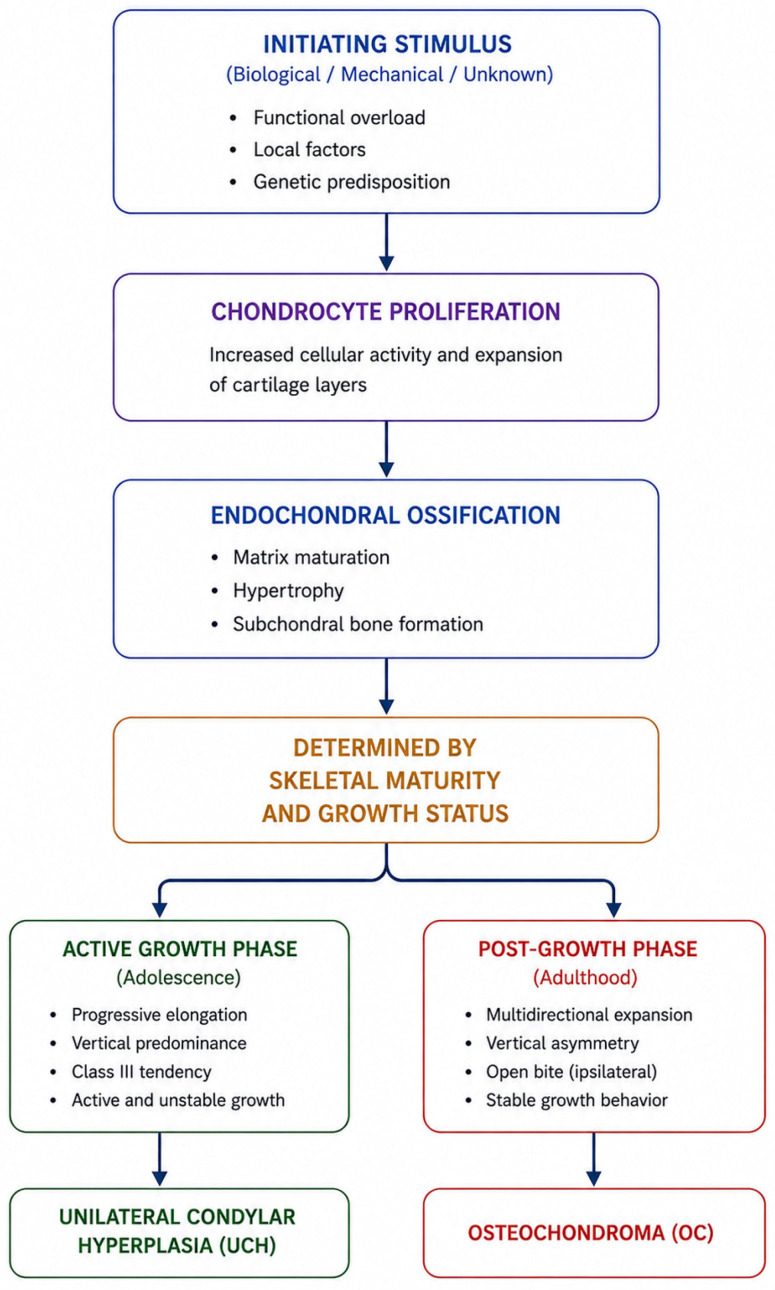
Biological and clinical determinants shaping the development and presentation of unilateral condylar hyperplasia and condylar osteochondroma. This schematic illustration was created using design software (Canva, online version) and subsequently processed and enhanced in ChatGPT (OpenAI) and was then reviewed and validated by the authors.

Overall, unilateral condylar hyperplasia and condylar osteochondroma appear to exhibit relevant histological similarities, including increased cartilage thickness, active chondrocyte proliferation, cartilage–bone structural continuity, and the involvement of anabolic growth factors (Figure 2). The observed differences may be primarily quantitative rather than qualitative, which could partially explain the diagnostic difficulty.

### 3.3. Role and Evolution of Proportional Condylectomy

Accumulated experience demonstrates that condylectomy is a primary treatment that provides long-term stability, particularly when combined with orthodontics to correct deformities [35]. In selected cases, the simultaneous approach of condylectomy and orthognathic surgery allows treatment of the disease while reducing total treatment time and achieving facial symmetry [54].

Different studies have reported heterogeneous clinical conditions, variable treatment approaches, and diverse methodological designs, resulting in significant variability among publications. In this context, the included summary table (Table 1) contains published studies involving different approaches, illustrating part of the heterogeneity regarding patient selection, surgical techniques, and reported outcomes.

Most of the currently available evidence regarding condylectomy and unilateral progressive condylar growth is based on retrospective studies and case series with relatively limited sample sizes. Long-term prospective multicenter studies remain necessary to further validate treatment protocols and outcomes.

Evidence regarding growth following early condylectomy shows that the mandible may continue to develop in a physiological manner without reproducing the previous asymmetry [63]. This concept has modified the traditional paradigm of waiting for the “burnt-out” phase before intervention, as delaying joint surgery allows asymmetry to continue progressing, altering both bone structure and soft tissues, including ligaments, muscles, and facial soft tissues.

Comparisons between patients treated during active versus inactive phases suggest that early control of growth reduces the magnitude of residual deformity, although final outcomes depend on the accumulated degree of structural asymmetry [64]. In contrast, exclusively conservative management has shown limitations in controlling progression in active cases [65].

Low condylectomy is commonly used in the management of OC [66], and in cases with significant deformities, it may be combined with simultaneous reconstructive techniques, including pedicled or free vertical osteotomies, repositioning of the residual condyle, or alloplastic reconstruction [67]. The combination of condylar resection and orthognathic surgery is also common in these cases [68].

A PubMed search using the term “proportional condylectomy” identified 25 publications up to January 2025. Fariña et al. [58] had previously described the concept of low condylectomy in selected cases of UCH, reporting encouraging clinical outcomes that provided clinical foundation for this surgical approach. Building on this experience, the same group later formally introduced the concept of proportional condylectomy in 2016 [4], proposing it as a structured surgical strategy for the management of UCH and the facial asymmetry.

Proportional condylectomy was defined as the selective resection of the hyperplastic condylar segment until structural symmetry is achieved between both condyle-ramus units (right and left), avoiding standardized high or excessive resections. The study by Fariña et al. [4] compared two groups with active UCH: one treated exclusively with high condylectomy (5 mm) and the other with proportional condylectomy. The results showed that approximately 80% of patients treated with high condylectomy required secondary orthognathic surgery, whereas only 20% of those treated with proportional condylectomy required subsequent orthognathic intervention. This finding demonstrated that individualized proportional resection could significantly modify therapeutic outcomes, differentiating it from the conceptual framework previously represented by the work of Wolford [3,55], in which high condylectomy was commonly combined with facial osteotomies and disk suturing.

In 2017, Mouallem et al. [69] published a series of 73 patients treated with proportional condylectomy, including cases managed between 1980 and 2015. This extended period introduced significant heterogeneity in imaging diagnostic methods and orthodontic and surgical protocols. Planning was primarily performed using two-dimensional frontal cephalometry, measuring the distance between the mandibular angle and the condyle. In that series, only 38% of patients were treated exclusively with condylectomy, while the majority required additional facial osteotomies. The mean age was 22 years, and significant variability in the extent of condylar osteotomy was observed.

As early as 1983, Delaire described the possibility of performing condylar osteotomies at different levels to harmonize the mandibular ramus in orthognathic surgery for patients with asymmetry, contributing technical versatility to the procedure. Subsequently, Araz et al. [70] reported two cases in which facial symmetry was achieved following condylectomy, suggesting the possibility of adapting the osteotomy level according to the deformity. This concept was later systematized by Nitzan in [71], who defined patient-specific adaptable condylectomy based on the type of facial deformity and magnitude of asymmetry, using dental study models to assess midline deviation and achieve occlusal symmetry.

The 2016 proposal by Fariña et al. [4], however, was based on an approach aimed at simultaneously treating active condylar disease and facial deformity without requiring complementary orthognathic surgery. This concept differed from previous approaches by positioning protocolized proportional condylectomy as a direct response to progressive asymmetric deformities, particularly effective in young and adolescent patients.

From 2019 onward, a phase of technical refinement continued. Sembronio et al. [72] studied seven patients using three-dimensional analysis to accurately determine the optimal level of condylar osteotomy, incorporating 3D-printed surgical guides to improve procedural accuracy. That same year, Niño-Sandoval et al. [14] published a systematic review and meta-analysis evaluating the role of proportional condylectomy in UCH. Using strict inclusion criteria, only two studies both published in 2015 and 2016 by the same group met the requirements for inclusion. The authors concluded that the technique may be valid and could potentially reduce the need for future surgeries. However, Brignardello-Petersen [73], in an analysis published in JADA, noted that the quality of evidence was low and insufficient to conclusively confirm its effectiveness in preventing secondary orthognathic surgery.

Abboud et al. [74] conducted a retrospective study of 14 adult patients treated exclusively with proportional condylectomy, concluding that the technique does not always achieve complete symmetry in adults and that a relevant proportion of patients may remain dissatisfied due to residual asymmetries in facial contour and mandibular base. They also emphasized that inclusion of younger patients in active growth may favor more stable outcomes.

**Figure 3:**
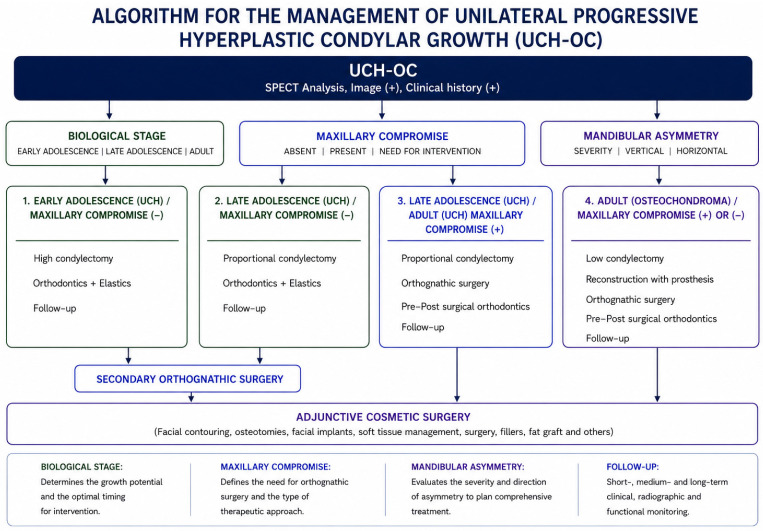
Clinical decision-making algorithm for treatment selection in unilateral progressive condylar growth. This schematic illustration was created using design software (Canva, online version) and subsequently processed and enhanced in ChatGPT (OpenAI), and was then reviewed and validated by the authors.

In the same year, Fariña et al. [75] published a three-dimensional analysis of 21 patients at different postoperative stages, demonstrating that at 12 months, condylar symmetry between both sides was achieved along with corticalization of the operated condyle. In the dentoalveolar component, variable areas of intrusion and extrusion were observed, highlighting the use of postoperative elastics as a strategy to achieve stable occlusion (Figure 3).

In 2020, Ha et al. [76] proposed specific guidelines for associated orthodontic management, attempting to standardize the therapeutic sequence. That same year, Hass et al. [77] described the intraoral approach for proportional condylectomy as a minimally invasive technique, particularly useful when combined with orthognathic surgery. Although the intraoral approach had not yet been described in early reports of the 1950s, surgical access to the mandibular condyle was initially reported using extraoral and transmeatal approaches [78,79], preceding the development of modern guide-assisted techniques.

Later, Sembronio et al. [80] reported five patients treated with proportional condylectomy combined with orthognathic surgery in a single surgical stage using virtual planning. Cascone et al. [81] described two patients treated exclusively with proportional condylectomy combined with disk fixation using anchors. In 2021, Cascone et al. [82] introduced “slice functional condylectomy,” based on multiple axial sections of the condyle to facilitate disk reinsertion. On other hand, Bussink et al. [83] incorporated augmented reality in an adult patient to use in proportional condylectomy.

van Bakelen et al. [84] demonstrated significant differences between three-dimensional measurements and panoramic radiographs in 32 patients, concluding that panoramic imaging is not suitable for precise analysis in proportional condylectomy. Cohen et al. [85] evaluated 15 patients treated without immediate postoperative orthodontics, observing early contralateral open bite that resolved spontaneously after six months; 94% reported esthetic satisfaction.

Abboud et al. [86] analyzed adult patients with hemimandibular hyperplasia type 2, confirming vertical improvements but highlighting limitations in severe asymmetries. Nitzan [71] described two differentiated protocols based on the magnitude of midline deviation, adapting the level of resection intraoperatively without disk suturing.

More recently, Sembronio et al. [62] compared intraoral and extraoral approaches, finding no significant differences in outcomes, despite emphasizing the steep learning curve associated with the intraoral technique. Cohen et al. [85] compared active UCH treated with proportional condylectomy versus inactive UCH treated with orthognathic surgery, demonstrating comparable results in stability and patient satisfaction. Perrotta et al. [87] analyzed and proposed a therapeutic protocol incorporating specific orthodontic models prior to proportional condylectomy. Finally, Tousidonis et al. [88] and Smolka et al. [89] reinforced the use of digital and titanium 3D-printed surgical guides as reproducible and precise tools.

### 3.4. Clinical Conditions for the Selection of Condylectomy and Surgical Treatment Sequence

Condylectomy represents the treatment of choice when progressive asymmetric condylar growth is present [69]. Undoubtedly, each patient must be treated individually, and any algorithm or therapeutic decision-making system must be adapted to the specific needs of each case. Even secondary cosmetic surgery may be required, considering that esthetics is a subjective condition dependent on the observer [90].

Postoperative analysis demonstrates a transient reduction in maximum mouth opening and alterations in muscular activity, with an initial deviation toward the operated side [45]. In this context, functional rehabilitation plays a relevant role in patient recovery.

From a clinical standpoint, three critical clinical elements define complexity of the treatment in unilateral progressive condylar growth: the volume and features of the condyle, the magnitude of mandibular asymmetry and the three-dimensional position of the maxilla. In some cases, prolonged progression of asymmetry in the lower third has led to secondary adaptation of the maxilla and its occlusion, such that asymmetry generated by UPCG affects not only the lower third but also the midface [91].

The maxilla, as a transitional structure between the upper third whose most significant growth occurs up to approximately 5 years of age (cranial base) and the lower third whose most pronounced growth occurs after 10 years, plays an articulating role between these segments, stabilizing facial structural morphology [92].

Sarnat [93] noted that nasal septum deviation is one of the most common anomalies even in individuals with normal growth, which may be associated with early stabilization of the upper third (ethmoid and vomer). However, maxillary adaptation, including final septal positioning, is also necessary for integration with the mandible. This hypothesis supports the dynamic interrelationship between maxillary and mandibular components in facial development.

According to our clinical analysis, when asymmetry presents with clinically significant maxillary involvement, treatment should be performed in conjunction with orthognathic surgery (bimaxillary surgery), as a predictable and reproducible strategy. In the absence of maxillary involvement, an exclusively mandibular or condylar approach represents the preferred sequence.

## 4. Discussion

Bone scintigraphy using SPECT and SPECT/CT has been widely applied in the diagnosis of unilateral condylar hyperplasia [94,95]. Several studies demonstrated good diagnostic performance, with reported sensitivities ranging from 81% to 95% and specificity values between 77% and 96%, particularly when quantitative uptake differences between condyles were analyzed [95,96]. SPECT/CT may improve anatomical evaluation; however, some studies highlighted potential disadvantages of SPECT/CT compared with conventional SPECT, including increased radiation exposure, higher costs, and limited additional diagnostic benefit despite slightly improved specificity values [96].

Current evidence indicates that SPECT and SPECT/CT should not be considered definitive methods for diagnosis. A recent meta-analysis including 887 patients demonstrated pooled sensitivities of 81.4% for SPECT and 81.8% for SPECT/CT, while pooled specificity reached 77.4% and 90.1%, respectively [96]. Despite these favorable values, important heterogeneity exists among studies regarding diagnostic thresholds, reference standards, follow-up protocols, and interpretation criteria [97]. Therefore, bone scintigraphy findings should always be interpreted together with clinical evaluation, serial imaging, morphological progression, and histopathological findings whenever available [96,97].

Age at diagnosis, as discussed in this review, represents a relevant factor in treatment definition. Early detection of condylar asymmetry and UCH allows identification of a progressive process. Olate et al. [59] reported initial results of condylectomy in patients at the late stage of mixed dentition (11 to 12 years old approximately), indicating that the permanent maxillary canine in eruption or early function represents a triggering variable for performing condylectomy combined with postoperative orthodontics using elastics.

This protocol was applied in nine patients, demonstrating high condylar regenerative capacity, adaptation of the new condyle, and occlusal optimization through the use of elastics, modifying critical scenarios that initially presented as Class III and evolved toward stable Class I relationships with balanced occlusion. Positive outcomes were reported at more than five years of follow-up. It is important to note that condylectomy in children and adolescents to prevent facial deformities was already described more than 50 years ago by Adler [98], introducing the technique as an option for managing early facial deformity.

Although the available evidence regarding early intervention remains limited and primarily based on retrospective case series, recent clinical guidelines have also emphasized the potential relevance of early surgical management in active condylar hyperplasia. The German S3 Clinical Practice Guideline on Condylar Hyperplasia highlighted that delayed treatment may lead to progressive facial deformity, more complex surgical correction, and increased functional impairment, while earlier intervention could potentially reduce secondary skeletal deformities and facilitate more favorable orthopedic and orthodontic adaptation [99]. The guideline additionally recognizes that current therapeutic decisions should be individualized according to growth activity, clinical progression, facial asymmetry, and functional impairment, particularly considering the still limited level of evidence available in the literature.

Anecdotally, in the report by Olate et al. [59], measurements between the hyperplastic and non-hyperplastic sides showed an average difference of approximately 5 mm, allowing proportional resections equivalent to a high condylectomy. Thus, in individuals aged 11 to 13 years, it is feasible to consider isolated high condylectomy combined with elastics and orthodontics to guide occlusion and stabilize facial condition.

When asymmetry remains progressive over time, with mandibular deviation, unilateral crossbite, and no significant maxillary growth alteration, the proposed treatment sequence is proportional condylectomy. This procedure not only addresses condylar disease but also mandibular deformity, as proportional resection allows mandibular repositioning based on the magnitude of asymmetry, achieving similarity in bilateral posterior vertical positioning [4,100].

The immediate clinical response following proportional condylectomy is the appearance of an open bite on the non-operated side. This occurs because the mandibular ramus and operated condyle reposition with vertical and posterior elevation, generating a three-dimensional mandibular change [7,59]. In most cases, orthodontics and elastics constitute the only necessary intervention to maintain the mandible centered relative to the maxilla, often achieving complete facial symmetry.

This situation is typically observed in adolescent patients between 14 and 18 or 19 years of age, who in most cases do not require a second surgical intervention, as previously demonstrated [4]. However, follow-up in these patients is essential, as some may still require secondary orthognathic surgery or complementary cosmetic procedures.

In patients with asymmetric maxillary involvement and progressive condylar growth, treatment usually consists of combined surgery involving proportional condylectomy associated with orthognathic surgery [6,101]. In these cases, the extent of resection is variable and depends on each clinical scenario. Our preference is to perform proportional condylectomy to homogenize posterior vertical asymmetry and reposition the mandibular ramus into a more symmetrical position relative to the contralateral side.

Regarding the use of sagittal split osteotomy of the mandibular ramus, most cases are performed bilaterally during the same surgical stage as condylectomy. Exceptions have been observed in cases where proportional resection fully corrects asymmetry on the affected side, eliminating the need for sagittal osteotomy on that side, while maintaining it on the non-hyperplastic side [102]. From our perspective, treatment based exclusively on proportional condylectomy is not efficient in cases of maxillomandibular deformity, and the indication for bimaxillary surgery is necessary.

Finally, in patients with significant condylar growth compatible with OC, it is necessary to consider not only low condylectomy but also reconstruction [40]. Cases of progressive condylar growth without associated mandibular or maxillary involvement are rare; therefore, the usual approach includes low condylar resection combined with orthognathic surgery when required [5].

Management of the resected area includes repositioning of the articular disk, reconstruction with vertical osteotomy, or the use of alloplastic prostheses as a contemporary and technologically advanced alternative for joint replacement [103]. This provides a predictable and stable sequence, allowing resolution of the pathology in a single surgical stage with reproducible outcomes.

In all cases, esthetic refinement is often necessary, and in some instances, a second procedure is required to improve facial contours and shape. For these purposes, the use of contour implants is typically the most appropriate option [104], although basal resection may also be performed in selected cases to optimize mandibular volume and contour. Genioplasty techniques also contribute to improving esthetic proportions and are particularly useful in cases of progressive asymmetry associated with unilateral condylar growth.

Inactive condylar growth, severe degenerative joint disease, or poorly defined diagnostic activity may represent limitations or relative contraindications for this approach. Furthermore, although proportional condylectomy has demonstrated favorable functional and esthetic outcomes in retrospective studies and clinical series, potential limitations and complications such as residual asymmetry, undercorrection, overcorrection, occlusal instability, and temporomandibular dysfunction must still be considered. Consequently, successful management requires adequate surgical experience, multidisciplinary planning, virtual surgical analysis, and careful postoperative orthodontic follow-up in order to optimize long-term stability and facial balance.

Based on these considerations, the proposed algorithm (Figure 3) integrates different therapeutic modalities in a structured manner, allowing clear interpretation and systematic clinical application in patients with UPCG.

The proposed clinical algorithm should be interpreted as a conceptual and clinically oriented decision-making model based on the currently available literature and the biological, imaging, and therapeutic considerations discussed throughout this review. Although the algorithm has not undergone prospective clinical validation or reliability assessment, current evidence and accumulated clinical experience suggest that it may represent a feasible strategic framework for organizing diagnostic integration and treatment planning in unilateral progressive condylar growth. Nevertheless, future prospective and multicenter studies remain necessary to evaluate its reproducibility, clinical applicability, and long-term validity.

## 5. Conclusions

Although proportional condylectomy has demonstrated favorable functional and esthetic outcomes in retrospective studies and clinical series, the currently available evidence remains limited by relatively small sample sizes, methodological heterogeneity, and the lack of prospective multicenter validation. Therefore, current conclusions should be interpreted with caution, and further standardized long-term investigations are necessary to better define the reproducibility, predictability, and long-term stability of this therapeutic approach.

Unilateral progressive condylar growth is a dynamic and multifactorial condition involving biological, skeletal, and functional components that directly influence diagnosis and treatment planning. Current evidence supports the concept that unilateral condylar hyperplasia and condylar osteochondroma may represent different manifestations of a related biological process. In this context, condylectomy represents a rational and individualized therapeutic strategy for controlling active condylar growth and improving mandibular asymmetry. The integration of clinical evaluation, three-dimensional imaging, and growth assessment remains essential for accurate decision-making.

## Figures and Tables

**Table 1:** Summary of representative studies on surgical management of UPCG.

Author/Year	Study Design	Sample Size	Main Surgical Approach	Outcome
Wolford et al. 2014 [55]	Retrospective study	37	Low condylectomy + orthognathic surgery	Improvement in facial symmetry and occlusal stability
Brusati et al. 2010 [56]	Case series	15	Condylectomy	Control of condylar hyperactivity and functional stability
Saridin et al. 2010 [57]	Case–control study	33	High condylectomy	More joint-related temporomandibular problems develop
Fariña et al. 2015 [58]	Retrospective case series	16	Low condylectomy + orthodontic management	Favorable esthetic and functional outcomes
Olate et al. 2023 [59]	Case series	9	Proportional condylectomy + orthodontic management	Stable postoperative symmetry and occlusion
Mehra et al. 2016 [60]	Retrospective study	21	Low condylectomy	Functional improvement and reconstruction using costochondral graft or tmj prosthesis
Wolford et al. [61]	Case–control study	42	High condylectomy + orthognathic surgery	Stability in tmj function and facial morphology
Sembronio et al. [62]	Descriptive comparative study	42	Proportional condylectomy with or without orthognathic surgery	Intraoral or preauricular approach comparison with good results with both techniques

## Data Availability

The data are available upon request from the corresponding author.
